# Pre-departure Training for Healthcare Students Going Abroad: Impact on Preparedness

**DOI:** 10.29024/aogh.2378

**Published:** 2018-11-05

**Authors:** A. Gatebe Kironji, Jacob T. Cox, Jill Edwardson, Dane Moran, James Aluri, Bryn Carroll, Chi Chiung Grace Chen

**Affiliations:** 1 Johns Hopkins School of Medicine, Baltimore, MD, US; 2 Department of Gynecology and Obstetrics, Johns Hopkins School of Medicine, Baltimore, MD, US

## Abstract

**Background:**

Many medical and nursing schools offer opportunities for students to participate in global health experiences abroad, but little is known about the efficacy of pre-departure training in preparing students for these experiences.

**Objectives:**

The primary aim was to identify characteristics of pre-departure training associated with participants’ reporting a high level of preparedness for their global health experiences. Secondary objectives included identifying students’ preferred subjects of study and teaching modalities for pre-departure training.

**Methods:**

A questionnaire was distributed to all medical and nursing students at our institution from 2013 to 2015. Questions addressed prior global health experiences and pre-departure training, preferences for pre-departure training, and demographic information.

**Findings:**

Of 517 respondents, 55% reported having a prior global health experience abroad, 77% of whom felt prepared for their experience. Fifty-three percent received pre-departure training. Simply receiving pre-departure training was not associated with perceived preparedness, but pre-departure training in the following learning domains was: travel safety, personal health, clinical skills, cultural awareness, and leadership. Perceiving pre-departure training as useful was also independently associated with self-reported preparedness. Students’ preferred instruction methods included discussion, lecture, and simulation, and their most desired subjects of study were travel safety (81%), cultural skills (87%), and personal health (82%).

**Conclusions:**

Incorporating travel safety, personal health, clinical skills, cultural awareness, and/or leadership into pre-departure training may increase students’ preparedness for global health experiences. Student perceptions of the usefulness of pre-departure training is also associated with self-reported preparedness, suggesting a possible “buy-in” effect.

## Introduction

According to a 2013 report, approximately 30% of U.S. medical students participated in at least one global health experience abroad (GHEA) prior to graduation [1]. Benefits of these experiences include learning to care for patients with illnesses uncommon in the U.S., building confidence in physical exam skills, and learning to work cross-culturally and with underserved populations [2,3,4]. These experiences also help to mold more socially conscious doctors and build a foundation for future careers in global health and primary care [2,5,6]. However, as GHEAs become increasingly popular, concerns have risen regarding the adequacy of student preparation, the ethical implications of these trips, and the potential harms posed to both patients and students [7]. Furthermore, GHEAs can become one-sided, unsustainable interactions between high- and low-resource institutions [8]. However, research suggests that many of these harms may be mitigated by comprehensive pre-departure training (PDT) for medical trainees conducting research and clinical electives abroad [5,8,9].

Calls for GHEA pre-departure training have come from both trainees and medical educators [5,10]. In one study conducted in Germany, the majority of medical students reported receiving insufficient preparation prior to departing abroad [11,12]. In Canada, the Canadian Federation of Medical Students (CFMS) has developed guidelines for PDT programs in response to these concerns [13]. Professional societies like the Association of Faculty of Medicine of Canada (AFMC) have adopted the same guidelines recommending PDT for medical students going abroad, and the United Kingdom’s Global Health Learning Outcomes Working Group has developed a list of competencies students should address prior leaving their home country [13,14]. In the US, the American Academy of Pediatrics (AAP) and the American College of Emergency Physicians (ACEP) have made similar recommendations, although they have focused on resident physicians rather than students [15,16].

Still, the adoption of PDT among GHEA programs remains low [17,18,19,20]. Recent surveys indicate that while the majority of US medical schools offer GHEAs to students, only a quarter to a third offer any training in global health prior to departure and fewer still make such training required [21,22]. While CFMS and AFMC recommend that PDT cover certain subjects (i.e. personal health, travel safety, cultural awareness, language, ethics), data regarding what is appropriate content for PDT for health professions students remain scarce [13,23]. Little is also known about which learning domains health professions students would like covered in PDT or in which format they would like these presented.

The primary objective of this study was to determine which training methodologies were associated with participants’ reporting a high level of preparedness for their GHEAs. Further analyses were run to assess for associations between student GHEA preparedness and any other demographic/experiential factors and student preparedness. Secondary objectives included identifying students’ preferred PDT learning domains, as well as their preferred training format for these sessions.

## Methods

We performed a cross-sectional, online survey to determine which medical and nursing students had participated in a GHEA and, of that group, how prepared they felt for their GHEA. All full-time medical and nursing students at Johns Hopkins University during academic years 2013–14 and 2014–15 received a survey link via a standardized email. We incentivized participation with a raffle for nominal-value gift cards. No unique participant identifiers were obtained, and the study was approved by the Johns Hopkins Hospital Institutional Review Board.

No validated survey tools were available to assess GHEA preparedness at the time of study. As such, our team performed a comprehensive literature review to inform development of a questionnaire addressing prior GHEAs, perceived preparedness for those GHEAs, desired PDT content and teaching modalities, and basic demographic information. This questionnaire was developed by a team of medical students and attending physicians at the Johns Hopkins University School of Medicine, all of whom have experience in GH. The preliminary questionnaire was then reviewed and refined using in-depth interviews with experts who were not involved in the initial questionnaire development, including a bio-ethicist, attending physicians, residents in several specialties, and medical students. This review process involved as series of standardized questions, including “What questions should be added to this survey?”, “What questions should be removed from this survey?”, and “Which questions should be rephrased in this survey and how?”. The review process also involved an open-ended, unscripted portion so that reviewers could provide feedback and suggestions that might not otherwise be broached by the standardized questions. The survey ultimately consisted of 41 multiple-choice and 5-point Likert scale items.

We administered the survey using Qualtrics (Qualtrics LLC, Provo, UT). Duplicate submissions and those in which students failed to answer principle questions (i.e. “Have you ever participated in any global health-related experiences that took place in another country?” or “Overall, how prepared do you think you were for your global health-related experience abroad?”) were removed. GHEA preparedness was addressed broadly with the question “Overall, how prepared do you think you were for your experience abroad?” The primary outcome was perceived preparedness for one’s prior GHEAs, as defined by a Likert score of 4 “*Prepared”* or 5 “*Very Prepared”*. Respondents with a Likert score of 1 “*Very Unprepared*”, 2 “*Unprepared*”, or 3 “*Neutral*” were characterized as *“Not Prepared”*. Continuous data were presented as means ± standard deviation (SD) and compared in the GH *“Prepared”* and *“Not Prepared”* groups using the Wilcoxon rank-sum test. Categorical data was presented as *n*-values with percentages and compared using the Chi-square test.

Unadjusted odds ratios (OR) with 95% confidence intervals (95% CI) were calculated on bivariate analysis comparing covariates and GHEA preparedness. Adjusted ORs with 95% CIs were calculated using multivariate logistic regression. A combination of forward and backward stepwise regression was used in the selection of the final model with *p*-value < 0.05 as the criterion for inclusion and *p*-value ≥ 0.05 as the criterion for exclusion. Regression models were further modified as needed based on Hosmer and Lemeshow’s goodness-of-fit testing and variance inflation factor (VIF). Data analysis was performed using Stata Statistical Software: Release 13 (Stata Corp, College Station, TX 2013). For analysis, age 27 years was selected as the cutoff for age dichotomization because (i) it was the median age of respondents and (ii) assuming high school graduation at age 18, “traditional” students who progress directly from college to medical/nursing school within the U.S. system would complete healthcare training by age 26 at the latest. Thus, the majority of respondents 27 years old or older likely qualify as “non-traditional students”, in the sense that they did not proceed directly to graduate school in nursing or medicine. (All Johns Hopkins nursing students must complete a bachelor’s degree prior to admission, thus their healthcare studies are strictly post-graduate.) Household income of $100,000/year was selected as the cutoff for household income dichotomization, because it (i) allowed for a relatively even distribution between groups and (ii) has been found by the Urban Institute to be the lower bound for upper-middle class within the U.S. [24] Eight weeks was selected as the cutoff for dichotomization of the duration of participants’ longest GHEA, as this was the median GHEA duration among participants and it is a cutoff used by some in the literature to define “short-term medical service trips” [25].

## Results

Of the 1,333 medical and nursing students who received the survey, 510 (38.3%) provided complete responses. The majority of respondents were female (76.2%) and in nursing school (58.6%), and the mean age was 27.6 years (range: 17–61) (Table 1). Household incomes were relatively evenly distributed, with those from households making $50,000–$100,000/year being most common (25.6%). Of those in medical school, the preponderance intended to enter a medical specialty (61.9%) rather than a surgical one (38.1%). Most participants (54.9%) had at least one prior GHEA. The only demographic factor significantly associated with having a prior GHEA was being female (*p*-value = 0.001).

**Table 1:** Respondent demographics and characteristics (*n* = 517).

Variable	Prior GHEA (*n* = 284)	No GHEA (*n* = 233)	*p*-value		Total

**Age, mean ± SD**	27.2 ± 5.0	28.0 ± 6.4	0.11		27.6 ± 5.6
**Gender, *n* (%)**					
Male	52 (42.3)	71 (57.7)	0.001	*	123 (23.8)
Female	232 (58.9)	162 (41.1)			394 (76.2)
**School Affiliation, *n* (%)**					
Medical School	113 (52.8)	101 (47.2)	0.41		214 (41.4)
Nursing School	171 (56.4)	132 (43.6)			303 (58.6)
**Annual Household Income, *n* (%)**					
<$50K	45 (57.7)	33 (42.3)	0.51		78 (20.4)
$50K–$99K	61 (62.2)	37 (37.8)			98 (25.6)
$100K–$149K	47 (59.5)	32 (40.5)			79 (20.6)
$150K–$199K	26 (65.0)	14 (35.0)			40 (10.4)
>$200K	45 (51.1)	43 (48.9)			88 (23.0)
**Intended Specialty, *n* (%)**^†^					
Medical	76 (60.8)	49 (39.2)	0.051		125 (61.9)
Surgical	36 (46.8)	41 (53.2)			77 (38.1)

Abbreviations: Global health experience abroad (GHEA), standard deviation (SD). Missing data for covariates: income (25.9%), intended specialty (5.6%). Surveys with no data regarding participation in prior GHEAs were excluded. ^†^M.D. candidates only. “Medical” = emergency medicine, family medicine, internal medicine, medical subspecialties, pediatrics, and psychiatry. “Surgical” = anesthesiology, general surgery, obstetrics/gynecology, and surgical subspecialties. *p-value < 0.05.

Of the respondents with a prior GHEA, most (56%) had participated in multiple GHEAs. The most common type of GHEA was clinical/service experiences (70%), with the frequency of research, international jobs, and study abroad being similar (36%, 36%, 28%, respectively). Roughly half (53%) had received PDT (Table 2). The most commonly covered PDT learning domains were travel safety (93%), cultural awareness (89%), and personal health (82%); these were also the most desired PDT learning domains. Those who received PDT generally considered it helpful (79%).

**Table 2:** Overview of Participants’ GHEAs (*n* = 284).

Variable	Total

**Number of GHEAs, *n* (%)**	
1	124 (43.7)
≥2	160 (56.3)
**Type of GHEA, *n* (%)**	
Research	102 (35.9)
Service/Clinical	199 (70.1)
Study Abroad	80 (28.2)
International Job	99 (34.9)
**Duration of Longest GHEA, *n* (%)**	
≤8wk.	144 (51.1)
>8wk.	138 (48.9)
**Pre-departure Training Received, *n* (%)**	
No	152 (53.5)
Yes	132 (46.5)
**Pre-departure Training Subjects, *n* (%)**^†^	
Safety	141 (92.8)
Health Precautions	125 (82.2)
Ethics	70 (46.1)
Clinical Skills	38 (25.0)
Research	38 (25.0)
Language	70 (46.1)
Culture	135 (88.8)
Leadership	52 (34.2)
**Helpfulness of Pre-departure Training, *n* (%)**	
Helpful	120 (78.9)
Not Helpful	32 (21.1)

^†^Percentages based on 152 respondents who reported receiving pre-departure training. Sum of percentages exceeds 100% because respondents’ pre-departure training may have covered multiple subjects.

Logistic regression found that simply receiving PDT was not significantly associated with student preparedness for GHEAs; however, 77% of those who received PDT did report feeling prepared for their experience. In an effort to determine which aspects of PDT were associated with students feeling prepared for GHEAs, bivariate logistic regression analyses were performed on the subset of participants who received PDT prior to going abroad (Table 3). This identified five PDT topics that were significantly associated with students feeling prepared: travel safety, personal health, clinical skills, cultural awareness, and leadership. The only other factors associated with perceived preparedness on bivariate analysis were reporting that PDT was useful and being a nursing student.

**Table 3:** Bivariate analysis for likelihood of feeling prepared (*n* = 152).

Variable	Crude OR		95% CI

**Sex**			
Male	Ref		
Female	1.32		0.50–3.48

**Age**			
<27	Ref		
>27	1.44		0.69–2.96

**Income**			
<$100,000/yr.	Ref		
>$100,000/yr.	1.16		0.38–3.55

**Intended Specialty**^†^			
Medical	Ref		
Surgical	0.74		0.22–2.54

**Number of Global Health Experiences Abroad**			
1	Ref		
≥2	1.26		0.58–2.71

**Current Degree Program**			
Medicine	Ref		
Nursing	2.41	*	1.10–5.27

**Do You Feel PDT Was Helpful?**			
No	Ref		
Yes	8.91	*	3.67–21.63

**PDT Topics Received**			
Safety	6.00	*	1.58–22.71
Health Precautions	2.79	*	1.15–6.74
Ethics	1.10		0.51–2.38
Clinical Skills	4.28	*	1.23–14.96
Research	0.73		0.31–1.71
Language	1.46		0.67–3.18
Culture	6.49	*	2.24–18.77
Leadership	3.07	*	1.18–7.99

Abbreviation: Pre-departure training (PDT). *p-value < 0.05. ^†^M.D. candidates only.

Using these results, a multivariate logistic regression model was run that controlled for the (i) inclusion in PDT of one or more of the five aforementioned learning topics that were significant for preparedness on bivariate analysis, (ii) whether or not PDT was perceived as useful, and (iii) the degree program. This found that receiving PDT in one or more of the topics that were mentioned above as being significant on bivariate analysis and reporting PDT as useful remained significantly associated with feeling prepared for GHEA (Table 4). However, there was no longer any significant difference between nursing and medical students.

**Table 4:** Multivariate analysis for likelihood of feeling prepared (*n* = 152).

Variable	Adjusted OR		95% CI

Current Degree Program			
Medicine	Ref		
Nursing	0.12		0.82–5.37
If PDT Received, Do You Feel It Was Helpful?			
No	Ref		
Yes	12.37	*	4.44–34.68
Inclusion of Culture, Safety, Clinical Skills, Leadership, and/or Health Precautions in PDT			
No	Ref		
Yes	8.47	*	2.21–32.52

Abbreviation: Pre-departure training (PDT). *p-value < 0.05.

**Figure 1:**
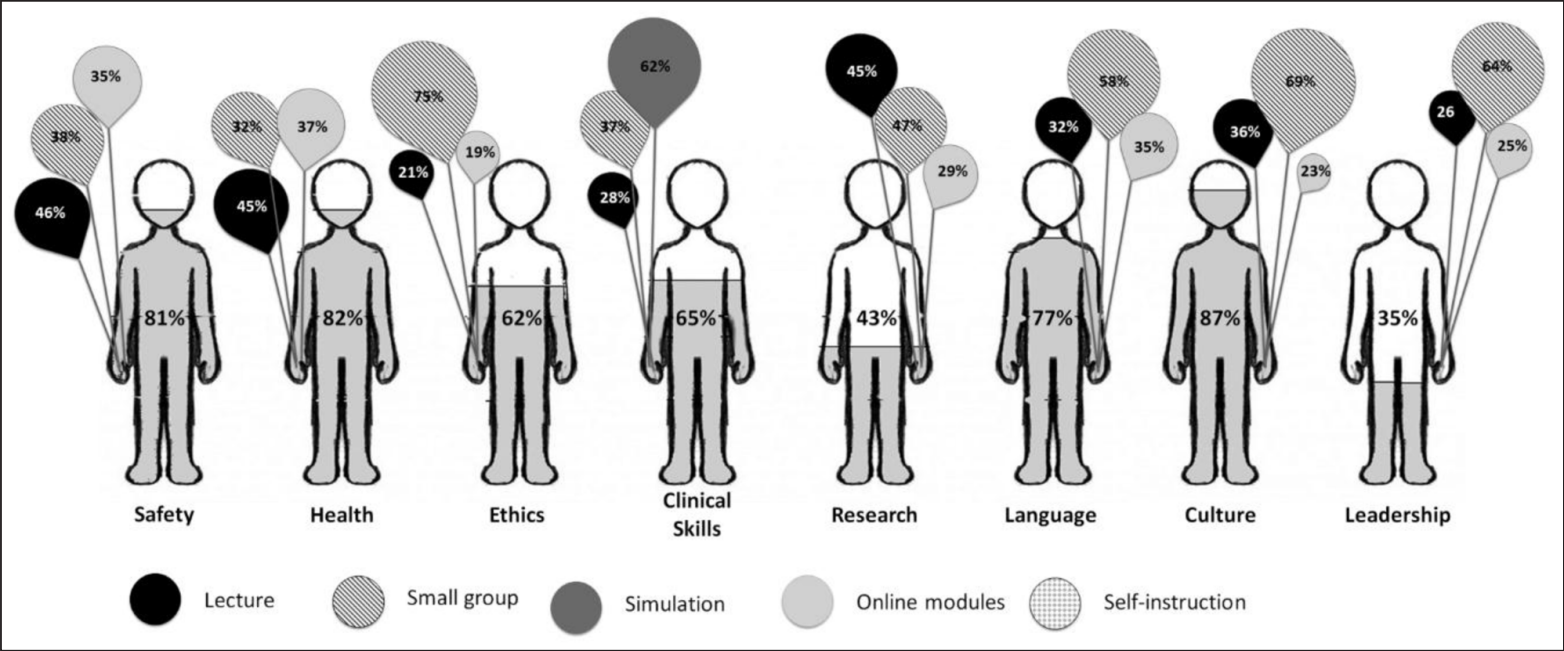
Pre-departure training topics that students preferred along with the preferred mode of instruction. The percentage of respondents that believed each pre-departure training topic as being important is represented by the people and the preference for a particular mode of instruction is represented by the balloons.

Among participants with prior PDT, the most commonly covered learning domains were safety (92.8%), cultural training (88.8%), and health precautions (82.2%) (Table 2). These were also the most desired PDT learning domains, with more than 80% of respondents indicating that these domains should be included in PDT (Figure 1). Research and leadership were the only learning domains that less than half of respondents perceived as important to include in PDT (43% and 35%, respectively). The most preferred format for PDT overall was small groups, but this varied by the topic. Students’ preferred mode of instruction was small groups for training in cultural skills and leadership, lectures for safety and health precautions, and simulations for clinical skills.

## Discussion

There was no significant association between simply receiving PDT and feeling prepared for GHEAs. However, certain aspects of PDT were found to be independently associated with an increase in perceived preparedness. This suggests that the efficacy of PDT varies depending on what is included in the training. The two PDT characteristics independently associated with feeling prepared were (i) the inclusion of select learning domains in the curriculum (i.e. travel safety, personal health, clinical skills, cultural awareness, leadership), and (ii) having students perceive their PDT as useful. With the association between these PDT characteristics and perceived preparedness now identified, we hope to explore why these associations may exist so that they can be leveraged to optimize future PDT programs.

It is worth noting that two of the five learning domains linked to perceived preparedness involve student wellbeing – namely, travel safety and personal health. Thus, it appears that PDT lessons in safety and personal health are not simply rehashing information that students already know and are instead providing new, useful information that will help participants stay safe and feel more comfortable in their host environment. Additionally, maximizing the safety of all involved should be the utmost priority of any GHEA program, and as such, programs should include travel safety and personal health training in PDT.

The significant association between clinical skills training and perceived GHEA preparedness may be explained by several factors. Firstly, the majority of participants (70%) had a clinical or service component to their GHEA, meaning that training in clinical skills had a clear relevance to their in-country work. Research has also shown that healthcare professionals in LMICS often overestimate the level of training and procedural capabilities of medical students visiting from developed nations [13,26] As such, many students returning from GHEAs report performing procedures with poor supervision while abroad and being asked to take on tasks that exceeded their training [8]. PDT that enables students to perform clinical tasks required in their host nation will not only help students feel more prepared, but may also improve their ability to contribute as members of the hosting healthcare team. Work by Margolick et al. indicates that the most important skills to teach medical students prior to GHEAs are: IV line insertion, suturing, assisting in surgery, post-operative wound management, and Foley catheterization, each of which would be appropriate for a medical student in the clerkship phase of his or her training [27]. However, any such training must also emphasize the importance of students only performing tasks for which they have been properly trained. Students may also need training in communication strategies for how to address situations in which they are asked to perform procedures outside their scope of practice.

The association between cultural awareness training and students feeling prepared is consistent with literature indicating that cross-cultural training is effective in preparing individuals for work experiences abroad [28]. The benefit of such training likely stems from many students not being familiar with the cultural norms of their host nation. Even brief cultural training for students may enable students to navigate cultural subtleties they might encounter in the field and thus engender respect and forge stronger relationships with host-country nationals [28]. Conversely, language training was not associated with participant preparedness, perhaps because PDT is generally too short for students to make significant enough advances in learning a language [29].

The association between leadership training and perceived GHEA preparedness is less easily explained than the other factors. It is possible that this may relate to student’s abilities to operate in team frameworks and understand the potentially hierarchical dynamics they may encounter while in some settings, but more research into this subject is warranted [30].

We should point out that while each of the aforementioned teaching domains was significant for students feeling prepared on bivariate analysis, they were not independently significant on multivariate analysis. Rather, what was significant for perceived preparedness on multivariate analysis was whether one or more of these topics had been included in students’ PDT. This may indicate that any one of these individual topics alone is not sufficient to noticeably improve students’ perceptions GHEA preparedness. However, of participants who received PDT in one of these domains, the majority (88%) had PDT in multiple relevant domains. Thus, it may be a cumulative effect wherein training in several of these subjects noticeably improves students’ sense of preparedness.

Notably, ethics, language, and research skills training were not associated with perceived preparedness. Regarding ethics, it may simply be that many medical and nursing students have already developed their own ethical standards and framework prior to medical/nursing school, and as such, ethics training may not significantly alter their approach to ethical issues faced on GHEAs. This is consistent with research indicating that PDT in ethics tended to instill less of a change in student behavior than PDT in any other domain [31]. Research skills training may have lacked significance due to the simple fact that most participants did not participate in research on their GHEA. As mentioned above, language PDT may show a limited benefit because PDT sessions are generally too brief to allow for significant linguistic improvement [29].

It is interesting to note that whether or not students perceived their PDT as useful was also independently associated with their perceived preparedness. There are several ways to explain this association. We hypothesize that this may be a reflection of the quality of students’ PDT programs. In other words, well-designed and well-executed PDT programs are more likely to be seen as useful and presumably also better prepare students for their GHEAs. However, this association may also represent a “buy-in” effect, wherein students who perceive their PDT as useful are more likely to fully engage in and learn from their training, and this in turn may also lead to better student preparedness; this is supported by research indicating that the most predictive factor of student learning is the amount of effort the instructor invests in making lessons engaging [32]. Alternatively, it may simply be that students who are inclined to perceive training as useful are also more inclined to perceive themselves as prepared for GHEAs than their counterparts.

Surprisingly, the number of prior international health experiences was not associated with perceived preparedness. It is unclear why this may be the case, but one possible explanation may be that participants who have been abroad multiple times are better able to realistically assess gaps in knowledge and skill level. Importantly, no demographic variables appeared to play a significant role in students’ sense of preparedness. This is encouraging because it suggests that factors that affect student perceptions of preparedness are largely modifiable.

This survey of nursing and medical students not only helps to highlight which PDT characteristics and learning domains are associated with self-reported GHEA preparedness, it also indicates which topics students prefer to learn about and their preferred modes of instruction for those topics. Adult learners are often focused on gaining specific knowledge and they require different approaches to meet their learning needs [33,34]. Our findings represent the students’ perspective, which is important for global health educators to consider when designing effective PDT.

Travel safety, personal health, ethics, clinical skills, language, and cultural awareness were learning domains that the majority of respondents felt should be included in PDT. While our findings indicated that many of these learning domains are associated with student preparedness, there were some exceptions: (i) leadership training was linked to perceived preparedness, but only 35% of students requested it; and (ii) the majority of students requested language (77%) and ethics PDT (62%), but these domains were not associated with perceived preparedness. The majority of participants with prior PDT experience reported only receiving training in travel safety, personal health, and cultural awareness. These results are consistent with prior findings that most programs include a limited number of learning domains in their PDT [11,20,35]. Canadian medical schools are a notable exception; one survey conducted in 2010 of all 17 Canadian medical schools found that PDT at nearly all the schools included travel safety, personal health, ethics, language, and cultural awareness [36]. Although our findings suggest that PDT can provide important preparation for GHEAs, further research is warranted to assess if these domains impact other outcomes such as student learning, burden on international partners, and patient outcomes.

The way a curriculum is structured and delivered has significant impacts on what students learn [33]. In our survey, students’ preferred mode of instruction varied by learning domain. Students in our study preferred ethics, cultural skills, and leadership to be taught in small groups. By allowing learners to share, criticize, and build on each other’s ideas, small groups offer a better environment for adult learners to learn, retain, and engage in higher-order thinking compared to didactic lectures [37]. In contrast, participants preferred a lecture format for travel safety and personal health topics; this may be because the material covered, such as how to get vaccinations or where to go for resources, is more suited for didactics rather than discussion. When it is difficult to have students physically present for this portion of PDT, such information can also be presented through online modules, which are effective when introducing learners to a broad range of easy-to-grasp topics [38]. For clinical skills, participants in our study overwhelmingly preferred a simulation format, thus allowing students to combine knowledge and procedural skills in a safe, controlled environment. Such simulations can help medical trainees become more comfortable with the diagnostic process, management of disease, and procedural skills [27,39,40].

One of the strengths of our study is the inclusion of both medical and nursing students in our survey. In addition, because we inquired about past GHEAs, our findings are reflective of the practices of other institutions as well as our own. One limitation of this study is the low response rate (44% for SOM and 36% for SON) and the risk of selection bias. We can speculate that it is likely that most of the students who did not respond to this questionnaire were either not interested in global health or had limited global health experience. Arguably this is less of a concern for our study as our target audience is trainees interested in global health. Our findings are also limited in that, to the best of our knowledge, there are currently no validated, objective measures of GHEA preparedness. As such, our preparedness data is based on self-reported, self-perceived preparedness. Such perceived-preparedness data is important, in that it presumably correlates roughly with true preparedness and in that students’ sense of being prepared is an important factor in their experience abroad. Nevertheless, developing a more objective measure of preparedness would be insightful and a worthwhile area for future work. Finally, our survey was retrospective in nature and could have been influenced by multiple factors, such as recall bias. We tried to minimize this by asking participants to give us details about their most recent experience.

We also feel that while increasing students’ perceptions of preparedness is one goal of PDT, it is not the only goal. Other objectives include ensuring appropriate patient care and fostering ethical, productive interactions between visiting and host communities. Thus, while our findings may serve to guide the development of future PDT curricula, they should not serve as the sole guidance for such development, particularly as these findings are preliminary and do not address PDT objectives aside from students’ perceived preparedness.

## Conclusions

Overall, this preliminary study of a convenience sample of medical and nursing students demonstrated that 55% of medical- and nursing-school participants had a previous GHEA, with 53% of those having received PDT. Participation in PDT that included sessions on travel safety, clinical skills, cultural awareness, leadership, and/or personal health was associated with a greater sense of preparedness for the GHEA. Perception of one’s PDT as useful was also linked to an increased sense of GHEA preparedness. As such, future PDT programs may benefit from incorporating the aforementioned learning domains and by maximizing student engagement. Most students with a prior GHEA and that received PDT thought that the topics of travel safety, personal health, cultural awareness, ethics, clinical skills, and language should be included in PDT. Moreover, students’ preferences regarding teaching format vary depending on the learning domain, such as small groups for ethics and simulation for clinical skills. Given the current lack of a standardized global health curriculum in the U.S. and other developed nations, the preliminary findings from this study can inform educators regarding the development of PDT to enhance future GHEAs with potential benefits for learners, international partners, and patients, though further research is warranted.

## Previous Presentations

Preliminary results were presented at the 2016 Annual CUGH Global Health Conference, April 2016, San Francisco, USA, and at the 2014 Annual CUGH Global Health Conference, May 2014, Washington DC, USA.

## Data Accessibility Statement

All data are readily available via email inquiry to the senior author, Dr. Chen, at cchen127@jhmi.edu.
